# Factors associated with mild cognitive impairment and dementia amongst the oldest old: findings based on the nationally representative “old age in Germany (D80+)” study

**DOI:** 10.1007/s40520-025-03022-7

**Published:** 2025-04-02

**Authors:** André Hajek, Razak M. Gyasi, Liang-Kung Chen, Karl Peltzer, Hans-Helmut König

**Affiliations:** 1 Department of Health Economics and Health Services Research, University Medical Center Hamburg-Eppendorf, Hamburg Center for Health Economics, Hamburg, Germany; 2 African Population and Health Research Center, Nairobi, Kenya; 3 National Centre for Naturopathic Medicine, Faculty of Health, Southern Cross University, Lismore, NSW Australia; 4 Center for Healthy Longevity and Aging Sciences, National Yang Ming Chiao Tung University, Taipei, Taiwan; 5 Center for Geriatrics and Gerontology, Taipei Veterans General Hospital, Taipei, Taiwan; 6 Taipei Municipal Gan-Dau Hospital, Taipei, Taiwan; 7 Department of Health Education and Behavioral Sciences, Faculty of Public Health, Mahidol University, Bangkok, Thailand; 8 Department of Psychology, University of the Free State, Bloemfontein, South Africa; 9 Department of Psychology, College of Medical and Health Science, Asia University, Taichung, Taiwan

**Keywords:** Mild cognitive impairment, Dementia, Cognitive decline, Oldest old, Aged, 80 and over

## Abstract

**Background/Aims:**

Particularly among the oldest old, there is restricted knowledge regarding the factors associated with mild cognitive impairment and dementia using data from large, nationally representative samples. Thus, our aim was to address this knowledge gap.

**Methods/Design:**

We used data from the nationally representative “Old Age in Germany (D80+)” study covering community-dwelling and institutionalized individuals in the entire country (*n* = 2,555). Mean age was 85.5 years (SD: 4.2), ranging from 80 to 100 years (61.7% of the participants were female). The DemTect was used to measure cognitive impairment in terms of probable mild cognitive impairment and probable dementia. Sociodemographic, lifestyle-related, psychosocial and health-related independent variables were included in the multinomial regression analysis.

**Results:**

In the analytic sample, 57.8% of the individuals did not have cognitive impairment, 24.2% of the individuals had mild cognitive impairment and 18.0% had probable dementia. Regression analysis identified some sociodemographic (e.g., advanced age, being male, lower education), lifestyle-related (lower cognitive activities), psychosocial (higher loneliness and absence of meaning in life), as well as health-related (e.g., functional impairment) factors associated with probable MCI and probable dementia. Loneliness was only associated with these outcomes among women, but not men.

**Discussions:**

Based on large, nationally representative data, this study revealed several factors associated with probable MCI and dementia – which enhances our current understanding mainly based on small or selective samples.

**Conclusion:**

Such knowledge may help to address those at risk for cognitive impairment. Longitudinal studies are required to gain further insights.

**Supplementary Information:**

The online version contains supplementary material available at 10.1007/s40520-025-03022-7.

## Introduction

High-income countries are undergoing significant demographic change and the number of older people, particularly those over 80, is steadily increasing. For example, in Germany, the proportion of the population aged 80 years and more was 7.2% in December of 2023 [1].

Previous research found that with increasing age, the likelihood of mild cognitive impairment (MCI) and dementia also increases [2]. Dementia is known as a progressive cognitive impairment syndrome mainly caused by Alzheimer’s disease. A previous meta-analysis showed a rather low prevalence of dementia (any type) amongst individuals 65 to 74 years (2.7%). This prevalence, however, markedly increases with age (e.g., 15.1% and 35.7% amongst individuals 80 to 89 years or 90 to 99 years, respectively) [2]. Another meta-analysis showed an overall prevalence of MCI of 16% among clinic and community populations [3]. High incidence rates for MCI were particularly observed among the oldest old (i.e., individuals aged 80 years and over) [4].

The more the disease progresses, the more difficult it becomes for individuals to manage their daily tasks - from complicated financial matters to basic activities such as washing. As a result, dementia patients require a high level of care and support [5], which can lead to nursing home placement [6] and premature mortality [7]. In addition, dementia represents a significant economic burden, as shown by the annual global societal cost of approximately USD 1313.4 billion for the 55.2 million people affected [8]. Given the multiple negative impacts of MCI and dementia, it is of great importance to explore the factors associated with them. This knowledge is crucial to clinicians/geriatricians, policymakers, and the overall well-being of dementia sufferers, their families and the health system.

The factors associated with MCI and dementia have been identified in several former studies (e.g [9, 10]. For example, a recent study demonstrated that advanced age and low education were risk factors for MCI among older Turkish individuals (mean age 74 years) [10]. In a further recent study, age and social relationships were associated with MCI based on a sample of older adults in Iran [11]. Another study showed, among other things, that men have a greater risk of MCI compared to women in oldest old Germans [9]. A further recent study also showed that several social and environmental factors (e.g., area deprivation or fine particulate matter) are associated with MCI based on community-dwelling adults 65 + in the United States [12].

Other studies investigated the determinants of dementia. For example, it was found that being male, advanced age, lower education, or stroke can increase dementia risk [13–15]. Based on data from UK Biobank, a recent study showed that, among other things, low self-rated overall health and a high multimorbidity risk score were associated with incident dementia among individuals with and without APOE4 [16]. Another work emphasized the association between metabolic factors such as high fasting blood glucose and dementia among South Asian countries [17].

Thus far, however, there is very restricted knowledge regarding the factors associated with MCI as well as dementia exclusively focusing on the *oldest old* based on *nationally representative samples* which also cover individuals living in *institutionalized surroundings* (which is important in order to include particularly vulnerable groups). The few existing studies exclusively using data from the oldest old, for example, were mainly limited to certain regions and only partly included individuals residing in institutionalized settings and are not fully generalizable to entire countries and very vulnerable groups [15, 18–20]. Thus, our aim was to investigate the factors associated with mild cognitive impairment and dementia using data from a large, nationally representative sample of the oldest old in Germany, including both community-dwelling and institutionalized individuals. This understanding is crucial for supporting individuals who are susceptible to MCI and dementia. Furthermore, it is important as maintaining cognitive function is paramount to successful ageing, especially in very advanced age [21].

## Materials and methods

### Sample

The data for this study came from the study “Old Age in Germany” (D80+), which is a comprehensive, nationally representative sample of individuals aged 80 and over in Germany who live both in the community and in nursing homes. The University of Cologne conducted the study in collaboration with ceres (Cologne Center for Ethics, Rights, Economics, and Social Sciences of Health) and the German Centre of Gerontology (DZA). The D80+ was financially supported by the Federal Ministry for Family Affairs, Senior Citizens, Women and Youth (BMFSFJ). Including both community-dwelling individuals and individuals residing in nursing homes allows for a more holistic understanding of individuals aged 80 years and over. Moreover, a sample reflecting both living arrangement enhances the generalizability of the findings.

Due to the pandemic, the study design was adapted and switched from face-to-face interviews to questionnaires (November 2020 to April 2021) and supplementary telephone interviews between May and October 2021. The written questionnaires focused on the essential content, while the telephone interviews covered topics of a bit lower priority. However, the cognitive function was a high priority. Nevertheless, for technical reasons it could not be carried out in questionnaires (but only in telephone interviews). Interview training took place for the telephone survey: three web training sessions (due to the pandemic) by infas project management (4 h each). The interviewers were also supervised for quality assurance purposes throughout the entire field phase. There was close contact with the project management.If relatives of a target person have informed the market research company responsible for data collection that the target person could not take part in the interview for health reasons, the relative was asked whether he or she could be contacted again for the telephone interview. A total of over 10,000 individuals took part in the survey, and more than 3,200 respondents have answered the contents (whereby the majority of the contents had a subordinate priority). In total, valid data on cognitive impairment was present for 2,922 individuals. Due to missings, the final analytic sample comprised *n* = 2,555 individuals. Further details regarding the methodology of the D80 + can be found here [22].

Respondents gave their consent to participate in this study. The D80 + study was approved by the Ethics Committee of the Medical Faculty of the University of Cologne (protocol number: 19-1387_1).

### Outcome: mild cognitive impairment and dementia

The DemTect screening instrument [23, 24] was used to assess probable mild cognitive impairment (MCI) and dementia. This measure yields a total score between 0 and 18, with higher scores indicating *less* cognitive impairment. Scores between 9 and 12 indicate MCI, while scores below 9 indicate dementia. Conversely, scores between 13 and 18 indicate no cognitive impairment. Previous studies have shown that DemTect can detect people with various dementias, including Alzheimer’s disease and vascular dementia, with a sensitivity of 85.1% and a specificity of 97%. It is also effective in identifying people with MCI, with a sensitivity of 80% and a specificity of 92% [23].

The Global Deterioration Scale (GDS) [25] was used in the proxy interviews. Here, relatives can rate the severity of cognitive deficits on seven levels. The GDS scale can also be divided into three categories: dementia (four to seven points), mild cognitive impairment (three points) and no cognitive impairment (one to two points) [26].

### Independent variables

Guides by previous research [13, 10], sociodemographic, lifestyle-related, psychosocial and health-related independent variables were included in regressions: Sociodemographic independent variables include sex (men or women), age group (80 to 84 years; 85 to 89 years; 90 years and over), marital situation (married; married, but living separately from spouse; divorced; widowed; single), education (ISCED-2011 classification [27], low, medium or high), and region (West Germany; East Germany).

With regard to lifestyle factors, we included the presence of sports activity (no; yes) and - similar to previous research [5, 28] count score for the number of eleven cognitive activities in the past 12 months (coffee wreath, café/restaurant, concert/theatre/museum, artistic activity (playing musical instruments), voluntary work, games (e.g., board games), further education (e.g., self-study or by attending lectures and courses), political events (e.g., from a party, or citizens’ initiative), receiving visitors (other than nursing or medical professionals), mental exercise (e.g., crossword puzzles or memory training), reading books; absence (0) or presence (1) in each case). The cognitive activities were then totalled, resulting in a score from 0 to 11 (with higher values reflecting a higher number of cognitive activities). Since previous research [29] emphasized the importance of modifiable lifestyle factors associated with cognitive impairment, we included sports activity and cognitive activities in our main model.

With regard to psychosocial independent variables, loneliness was measured using a single-item – which is well in line with other studies [30]. It ranges from 1 (reflecting “never or almost never”) to 4 (“always or almost always”) and explicitly refers to the past week. Higher values reflect higher loneliness levels. Value of life was quantified using an item with a high face validity explicitly referring to the meaning in life (no; neither; yes).

Regarding health-related factors, we focused on self-rated health (single item tool, from 1 = very poor to 4 = very good), functional impairment and chronic conditions.

Functional impairment was measured using a modified version of the Instrumental Activities of Daily Living (IADL) tool which was created by Lawton and Brody [31]. This tool has seven items. Rated on a scale from 0 (requiring assistance) to 2 (no assistance needed), each item refers to specific domains (e.g., preparation of meals or managing financial issues). The scores across all items were averaged. Thereafter, we reversed the coding. The final score thus ranges from 0 to 2 whereby higher scores reflect higher functional impairment. In a robustness check, we replaced this IADL tool with an ADL tool [32], 7-items; final score ranges from 0 to 2, whereby higher scores reflect higher impairment. In a further sensitivity analysis, functional impairment was removed from the model since functional impairment can also be a central accompanying symptom and consequence of dementia.

Cronbach’s alpha was 0.91 in this study. Following the multimorbidity index in old age [33, 34], the count of chronic diseases includes the presence (1) or absence (0) of 21 specific chronic diseases (e.g., joint or bone disease, heart failure, respiratory or lung disease).

For descriptive purposes, we also reported living arrangements (private living or being institutionalized). It is worth emphasizing that living arrangement was not included in the regression analysis as it could reflect the consequence of dementia rather than the cause (even if the degree of dementia could be exacerbated by entering a nursing home).

### Statistical analysis

First, the characteristics of the sample are categorised according to the status of cognitive impairment (individuals without cognitive impairment, individuals with probable MCI, individuals with probable dementia). Subsequently, multinomial logistic regressions were performed, which included the categories of individuals without cognitive impairment, individuals with probable MCI and individuals with probable dementia. Cluster-robust standard errors were computed. Sampling weights were applied in this study to tackle non-response and the disproportionate sampling design [22]. The weighted distributions correspond to the German microcensus.

Of note that individuals excluded from the analytical sample due to missing data did not significantly differ from individuals in terms of key factors such as sex (Cramer’s V = − 0.02, *p* = 0.21), age group (Cramer’s V = 0.03, *p* = 0.14), marital status (Cramer’s V = 0.03, *p* = 0.18) or self-rated health (Cohen’s d = − 0.01, *p* = 0.81).

Statistical significance was set at a p-value of ≤ 0.05. All statistical analyses were performed using Stata 18.0 (Stata Corp., College Station, Texas).

## Results

### Sample characteristics

For the analytic sample, characteristics of the sample stratified by cognitive impairment are presented in Table 1. Mean age was 85.5 years (SD: 4.2), ranging from 80 to 100 years (61.7% of the participants were female). Overall, 57.8% of the individuals did not have cognitive impairment, 24.2% of the individuals had mild cognitive impairment and 18.0% had probable dementia. Among individuals with probable dementia, about 31.1% were institutionalized, whereas 2.7% were institutionalized among individuals without cognitive impairment.

**Table 1:** Sample characteristics (analytic sample) by cognitive impairment status

	Not being cognitively impaired *N* = 1477	Probable mild cognitive impairment *N* = 617	Probable dementia *N* = 460
Sex: N (%)			
Men	522 (35.3%)	290 (46.9%)	169 (36.7%)
Women	956 (64.7%)	328 (53.1%)	291 (63.3%)
Age: N (%)			
80 to 84 years	1028 (69.6%)	299 (48.4%)	190 (41.4%)
85 to 90 years	344 (23.3%)	177 (28.7%)	168 (36.4%)
90 years and over	104 (7.1%)	142 (22.9%)	102 (22.2%)
Marital status: N (%)			
Married	641 (43.4%)	225 (36.5%)	135 (29.4%)
Married, living separated from spouse	14 (0.9%)	1 (0.2%)	0 (0.1%)
Divorced	70 (4.8%)	21 (3.4%)	15 (3.3%)
Widowed	681 (46.1%)	351 (56.8%)	295 (64.1%)
Single	70 (4.8%)	19 (3.1%)	14 (3.1%)
Living situation: N (%)			
Private living	1437 (97.3%)	540 (87.5%)	317 (68.9%)
Being institutionalized	40 (2.7%)	77 (12.5%)	143 (31.1%)
Education (ISCED-2011): N (%)			
Low	257 (17.4%)	151 (24.5%)	173 (37.5%)
Medium	754 (51.0%)	305 (49.4%)	215 (46.6%)
High	467 (31.6%)	161 (26.1%)	73 (15.8%)
Region: N (%)			
West Germany	1154 (78.1%)	467 (75.6%)	348 (75.6%)
East Germany	324 (21.9%)	151 (24.4%)	112 (24.4%)
Presence of sports activity: N (%)			
No	457 (30.9%)	285 (46.1%)	272 (59.2%)
Yes	1020 (69.1%)	333 (53.9%)	188 (40.8%)
Count score of cognitive activities (from 0 to 11 cognitive activities)	4.7 (1.8)	3.7 (1.8)	3.4 (1.6)
Loneliness (from 1 = never/almost never to 4 = almost or almost always, whereby higher values reflect higher loneliness): Mean (SD)	1.5 (0.6)	1.7 (0.8)	1.9 (0.8)
Meaning in life: N (%)			
No	39 (2.6%)	27 (4.4%)	74 (16.0%)
Neither	150 (10.1%)	83 (13.5%)	109 (23.8%)
Yes	1289 (87.2%)	507 (82.1%)	277 (60.3%)
Self-rated health (ranging from 1 = very bad to 4 = very good): Mean (SD): Mean (SD)	2.7 (0.6)	2.6 (0.6)	2.4 (0.6)
Count score of chronic conditions (based on 21 chronic conditions)	4.5 (2.6)	4.8 (2.6)	5.3 (2.7)
Functional impairment (IADL; ranging from 0 to 2, whereby higher values reflect higher functional impairment): Mean (SD)	0.3 (0.5)	0.6 (0.6)	1.2 (0.8)

Moreover, there were, among other things, quite large differences (Cohen’s d=|0.74|) in the cognitive activities and loneliness scores (Cohen’s d=|0.61|) between individuals without cognitive impairment and individuals with probable dementia. Very large differences (Cohen’s d=|1.54|) were observed in functional impairment between individuals without cognitive impairment and individuals with probable dementia. Please see Table 1 for further details.

### Regression analysis

In Table 2, the results of multinomial logistic regressions among the total sample are displayed with the base outcome of individuals without cognitive impairment. In comparison to individuals without such cognitive impairment, individuals having probable MCI were more likely to be male (RRR: 0.22, 95% CI: 0.15 to 0.34), being older (e.g., 90 years and older compared to 80 to 84 years, RRR: 4.27, 95% CI: 2.36 to 7.74), having a lower education (high education compared to low education, RRR: 0.55, 95% CI: 0.35 to 0.86), lower cognitive activities (RRR: 0.80, 95% CI: 0.74 to 0.86), having higher loneliness levels (RRR: 1.44, 95% CI: 1.17 to 1.76), and be more functionally impaired (RRR: 2.01, 95% CI: 1.43 to 2.82).

**Table 2:** Factors associated with probable mild cognitive impairment and probable dementia. Findings of multinomial logistic regressions (base outcome: individuals not being cognitively impaired) among the total sample

Independent variables	Probable mild cognitive impairment	Probable dementia
Sex: Women (Ref.: Men)	0.22***	0.30***
	(0.15–0.34)	(0.15–0.59)
Age group: − 85 years to 89 years (Ref.: 80 to 84 years)	1.92**	1.69*
	(1.25–2.94)	(1.00–2.84)
− 90 years and over	4.27***	1.41
	(2.36–7.74)	(0.69–2.89)
Marital status: Married, living together with spouse/Divorced/Widowed/Single (Ref.: Married)	1.16	0.99
	(0.86–1.58)	(0.61–1.61)
Educational level (ISCED-2011): - medium (Ref.: low)	0.67+	0.47***
	(0.45–1.00)	(0.31–0.72)
- high	0.55**	0.29***
	(0.35–0.86)	(0.16–0.54)
Region: East Germany (Ref.: West Germany)	1.23	1.22
	(0.92–1.65)	(0.78–1.92)
Presence of sports activity: Yes (Ref.: No)	0.90	0.99
	(0.69–1.17)	(0.67–1.45)
Count score of cognitive activities (from 0 to 11 cognitive activities)	0.80***	0.81***
	(0.74–0.86)	(0.74–0.89)
Loneliness (from 1 = never/almost never to 4 = almost or almost always, whereby higher values reflect higher loneliness)	1.44***	1.37*
	(1.17–1.76)	(1.08–1.75)
Meaning in life: - Neither (Ref.: No)	0.94	0.51+
	(0.43–2.06)	(0.23–1.10)
- Yes	0.96	0.32***
	(0.51–1.81)	(0.17–0.62)
Self-rated health (ranging from 1 = very bad to 4 = very good)	1.14	1.52**
	(0.91–1.43)	(1.13–2.05)
Count score of chronic conditions (based on 21 chronic conditions)	0.99	0.97
	(0.93–1.04)	(0.91–1.04)
Functional impairment (IADL; ranging from 0 to 2, whereby higher values reflect higher functional impairment)	2.01***	6.80***
	(1.43–2.82)	(4.61–10.04)
Constant	0.84	0.41
	(0.27–2.55)	(0.10–1.59)
Observations	2,555	
Pseudo-R²	0.20	

Relative risk ratios are shown; 95% CI in parentheses; *** *p* < 0.001, ** *p* < 0.01, * *p* < 0.05; sampling weights were applied (also adjusted for sample cells, which are utilized for the stratification of the secondary sampling unit)

In comparison to individuals without cognitive impairment, individuals having probable dementia were more like to be male (RRR: 0.30, 95% CI: 0.15 to 0.59), being older (85 to 89 years compared to 80 to 84 years, RRR: 1.69, 95% CI: 1.00 to 2.84), having a lower education (e.g., high education compared to low education, RRR: 0.29, 95% CI: 0.16 to 0.54), lower cognitive activities (RRR: 0.81, 95% CI: 0.74 to 0.89), absence of meaning in life (presence vs. absence, RRR: 0.32, 95% CI: 0.17 to 0.62), having better self-rated health (RRR: 1.52, 95% CI: 1.13 to 2.05), and be more functionally impaired (RRR: 6.80, 95% CI: 4.61 to 10.04).

The sex-specific multinomial regressions (with the same base outcome) are shown in Table 3. Mostly, findings were comparable between women and men. However, there were some interesting gender-specific findings. For example, higher loneliness levels were associated with higher chances of belonging to the probable MCI or probable dementia group (compared to the group without cognitive impairment) among women, but not men. Moreover, higher age was associated with higher chances of belonging to the probable MCI group (compared to the group without cognitive impairment) among women, but not men. More details are shown in Table 3.

**Table 3:** Factors associated with probable mild cognitive impairment and probable dementia. Findings of multinomial logistic regressions (base outcome: individuals not being cognitively impaired) – stratified by sex

Independent variables	Probable mild cognitive impairment – among men	Probable dementia – among men	Probable mild cognitive impairment – among women	Probable dementia – among women
Age group: − 85 years to 89 years (Ref.: 80 to 84 years)	0.97	1.44	1.94**	1.68+
	(0.66–1.43)	(0.88–2.36)	(1.26–2.97)	(0.94–3.01)
− 90 years and over	1.10	2.34*	5.00***	0.87
	(0.63–1.94)	(1.21–4.52)	(2.74–9.14)	(0.40–1.90)
Marital status: Married, living together with spouse/Divorced/Widowed/Single (Ref.: Married)	1.26	1.47	1.11	0.77
	(0.85–1.88)	(0.85–2.56)	(0.69–1.76)	(0.43–1.38)
Educational level (ISCED-2011): - medium (Ref.: low)	0.62	0.28**	0.60*	0.47**
	(0.25–1.57)	(0.11–0.69)	(0.38–0.94)	(0.27–0.81)
- high	0.36*	0.20**	0.82	0.24**
	(0.14–0.91)	(0.08–0.53)	(0.47–1.44)	(0.10–0.59)
Region: East Germany (Ref.: West Germany)	1.36	0.67	1.11	1.85*
	(0.92–2.01)	(0.36–1.24)	(0.71–1.73)	(1.01–3.41)
Presence of sports activity: Yes (Ref.: No)	0.85	1.36	0.97	0.78
	(0.60–1.19)	(0.83–2.25)	(0.65–1.44)	(0.44–1.36)
Count score of cognitive activities (from 0 to 11 cognitive activities)	0.86**	0.77***	0.75***	0.83*
	(0.78–0.95)	(0.69–0.87)	(0.67–0.83)	(0.71–0.97)
Loneliness (from 1 = never/almost never to 4 = almost or almost always, whereby higher values reflect higher loneliness)	0.88	1.03	1.73***	1.63**
	(0.66–1.19)	(0.70–1.53)	(1.32–2.27)	(1.16–2.29)
Meaning in life: - Neither (Ref.: No)	0.48	0.52	1.10	0.62
	(0.16–1.41)	(0.16–1.70)	(0.41–2.95)	(0.25–1.55)
- Yes	0.47+	0.32*	1.22	0.35**
	(0.19–1.15)	(0.11–0.96)	(0.54–2.77)	(0.16–0.78)
Self-rated health (ranging from 1 = very bad to 4 = very good)	0.97	0.87	1.33	2.00***
	(0.71–1.31)	(0.58–1.32)	(0.94–1.88)	(1.35–2.96)
Count score of chronic conditions (based on 21 chronic conditions)	0.99	1.13*	1.00	0.87**
	(0.91–1.07)	(1.02–1.24)	(0.92–1.08)	(0.79–0.96)
Functional impairment (IADL; ranging from 0 to 2, whereby higher values reflect higher functional impairment)	2.25**	2.14**	1.66*	14.71***
	(1.34–3.79)	(1.29–3.55)	(1.10–2.50)	(8.54–25.34)
Constant	4.49+	3.50	0.10**	0.04***
	(0.92–21.91)	(0.47–25.99)	(0.02–0.46)	(0.01–0.26)
Observations	1,321		1,234	
Pseudo-R²	0.12		0.30	

Relative risk ratios are shown; 95% CI in parentheses; *** *p* < 0.001, ** *p* < 0.01, * *p* < 0.05; sampling weights were applied (also adjusted for sample cells, which are utilized for the stratification of the secondary sampling unit)

In sensitivity analysis, IADL was replaced by ADL (Supplementary Table 1). However, findings remained nearly the same in terms of effect size and significance. Moreover, we removed functional impairment from our model (Supplementary Table 2). However, findings remained similar; mainly, some age effects were a bit more pronounced in terms of effect size and significance. In another model, the lifestyle factors were also excluded from the model (in addition to the functional impairment; see Supplementary Table 3), producing similar results compared to Supplementary Table 2.

## Discussion

Based on nationally representative data of individuals aged 80 years and over in Germany, our purpose was to identify the factors associated with probable MCI and probable dementia. We identified some sociodemographic (e.g., advanced age, being male, lower education), lifestyle-related (lower cognitive activities), psychosocial (higher loneliness and absence of meaning in life), as well as health-related (e.g., functional impairment) factors associated with probable MCI and probable dementia. In particular, it was only among women that loneliness was associated with these outcomes.

Previous studies already demonstrated an association of sociodemographic factors such as older age, having a lower educational level, and being male with MCI [9, 10] as well as dementia [13–15]. Age itself may not be a risk factor for the outcomes, but rather age-related factors such as declining overall health and changes in the immune system [35]. We assume that gender-specific risk factors, which include lifestyle factors and various medical conditions, may explain the higher risk of MCI and dementia in men [36]. A former meta-analysis also showed an association between lower education and a higher risk of dementia [37]. This may be attributed to cognitive reserves. More precisely, higher education may stimulate rises in cognitive reserves (i.e., improved adaptability of the brain to counteract neuropathological and vascular damage before the onset of dementia symptoms) [37].

Regarding lifestyle-related factors, our study demonstrated a negative association between cognitive activities and probable MCI and probable dementia. A prior systematic review and meta-analysis emphasized the association between cognitively stimulating activities and dementia [38]. One may assume that such activities can improve cognitive reserve [39] which may be negatively associated with dementia.

With regard to psychosocial factors, previous systematic reviews and meta-analyses (e.g [40, 41], noted that there was limited evidence regarding the association between loneliness and MCI, whereas there was a rather clear association between loneliness and dementia. In our study, loneliness was associated with both probable MCI and probable dementia. This may be explained as follows: Loneliness may be associated with reduced social activities, such as interpersonal and physical activities which can contribute to cognitive decline [42]. Other studies have also emphasized the role of loneliness in general self-worth which in turn can contribute to cognitive impairment [43]. Genetic factors may also explain the association between loneliness and dementia [44]. While previous studies are mainly based on older adults in general (e.g., 50 or 60 + years; community-dwelling; as an overview: [40, 41], our present study extends this knowledge by showing an association of loneliness with MCI and dementia based on a nationally representative sample of community-dwelling and institutionalized individuals aged 80 years and over in Germany (including a significant proportion of vulnerable groups such as individuals aged 90 and over, individuals with functional impairments or individuals with multiple chronic illnesses).

Interestingly, meaning in life was negatively associated with probable dementia in our study. A recent meta-analysis also demonstrated a robust association between meaning and purpose in life with reduced likelihood of developing dementia [45]. Factors that may explain this link are as follows (as an overview: [45]): Individuals who report greater meaning in life tend to have better health and engage in more health-promoting behaviours, potentially protecting cognitive function as they age. In addition, these individuals often exhibit higher levels of social integration and engagement in activities such as volunteering, which also can contribute to protective cognitive outcomes.

With regard to health-related independent variables: The association between increased functional impairment and the outcomes observed in our study is consistent with previous findings [46, 35]. These findings are highly reasonable as significant functional decline is recognised as a feature of dementia: early difficulties in more complex instrumental activities of daily living (IADL) may indicate progressive neurodegenerative processes that eventually lead to clinically detectable dementia [35].

Surprisingly, *favorable* self-rated health and a *lower* number of chronic conditions were positively associated with probable dementia (compared to not being cognitively impaired) among women. A previous study also found an association between the absence of multimorbidity and dementia – they attributed it to efforts (e.g., changes in lifestyle-related factors) to avoid subsequent cognitive impairment [9]. The association between favorable self-rated health and probable dementia among women may be attributed to the fact that they may compare their health to individuals worse off in the health domain [47]. This comparison may lead to favorable health evaluations which potentially may not reflect their “true” health status. This could lead to a neglect of lifestyle-related variables (e.g., more sedentary lifestyle) that could increase the probability of dementia. However, this is a speculative explanation requiring further testing in upcoming studies. Moreover, longitudinal studies are needed.

Several strengths and limitations warrant acknowledgment in this study. First, our study exclusively focused on the neglected group of individuals aged 80 years and above in a high-income country context. Moreover, we used data from a large, nationally representative sample encompassing both those residing in private households and those in institutionalized settings. Furthermore, individuals and their proxies (if needed) were interviewed. Established tools were employed to quantify explanatory variables (e.g., the tool used to quantify functional impairment), and cognitive impairment was assessed using a screening tool. Clinical diagnosis is the gold standard, and such screening tools can only give an indication of possible cognitive impairment.

However, great caution is recommended in interpreting the directionality of findings since this study is cross-sectional in nature. For example, dementia may also cause reductions in meaning in life. Future longitudinal studies are strongly recommended to disentangle such complex relationships. Of note that the response rate for the D80 + was modest. However, this must be seen in context (e.g. questionnaires and additional telephone survey of individuals aged 80 and over -, living at home or in institutions - during the pandemic). Appropriate sampling weights were also applied to address non-response and the disproportionate sampling design. 

In conclusion, based on large, nationally representative data, this study revealed several factors associated with probable MCI and dementia – which enhances our current understanding mainly based on somewhat restricted samples. Future longitudinal studies are urgently needed.

## Electronic supplementary material

Below is the link to the electronic supplementary material.

Supplementary Material 1

## Data Availability

No datasets were generated or analysed during the current study.
