# Supplementary material for: Factors associated with mild cognitive impairment and dementia amongst the oldest old: findings based on the nationally representative “old age in Germany (D80+)” study
Source: Aging Clin Exp Res. 2025 Apr 2;37(1):110. doi: 10.1007/s40520-025-03022-7 (PMC11965201; doi:10.1007/s40520-025-03022-7)
Supplement: Supplementary file 1 — Supplementary Material 1 [file 40520_2025_3022_MOESM1_ESM.docx]

Supplementary Table 1. Factors associated with probable mild cognitive impairment and probable dementia. Findings of multinomial logistic regressions (base outcome: individuals not being cognitively impaired) – with ADL

| Independent variables | Probable mild cognitive impairment | Probable dementia | Probable mild cognitive impairment – among men | Probable dementia – among men | Probable mild cognitive impairment – among women | Probable dementia – among women |
| --- | --- | --- | --- | --- | --- | --- |
|  |  |  |  |  |  |  |
| Sex: Women (Ref.: Men) | 0.23*** | 0.35** |  |  |  |  |
|  | (0.15–0.35) | (0.17–0.69) |  |  |  |  |
| Age group: − 85 years to 89 years (Ref.: 80 to 84 years) | 2.02** | 1.87* | 1.06 | 1.54+ | 2.02** | 2.00* |
|  | (1.33–3.07) | (1.12–3.14) | (0.72–1.55) | (0.94–2.52) | (1.33–3.08) | (1.16–3.44) |
| − 90 years and over | 4.66*** | 1.89+ | 1.28 | 2.70** | 5.12*** | 1.47 |
|  | (2.59–8.39) | (0.93–3.84) | (0.75–2.18) | (1.45–5.04) | (2.85–9.19) | (0.69–3.13) |
| Marital status: Married, living together with spouse/Divorced/Widowed/Single (Ref.: Married) | 1.14 | 0.97 | 1.23 | 1.45 | 1.10 | 0.72 |
|  | (0.84–1.55) | (0.59–1.58) | (0.84–1.81) | (0.84–2.52) | (0.69–1.76) | (0.40–1.28) |
| Educational level (ISCED-2011): - medium (Ref.: low) | 0.66* | 0.44*** | 0.63 | 0.28** | 0.58* | 0.41*** |
|  | (0.45–0.97) | (0.29–0.66) | (0.25–1.56) | (0.12–0.67) | (0.37–0.91) | (0.24–0.69) |
| - high | 0.54** | 0.27*** | 0.36* | 0.20** | 0.83 | 0.20*** |
|  | (0.35–0.83) | (0.15–0.48) | (0.14–0.90) | (0.08–0.52) | (0.48–1.43) | (0.08–0.50) |
| Region: East Germany (Ref.: West Germany) | 1.21 | 1.42 | 1.33 | 0.64 | 1.06 | 2.48** |
|  | (0.91–1.60) | (0.92–2.18) | (0.91–1.94) | (0.35–1.18) | (0.68–1.64) | (1.42–4.33) |
| Presence of sports activity: Yes (Ref.: No) | 0.92 | 0.94 | 0.82 | 1.30 | 1.01 | 0.82 |
|  | (0.70–1.19) | (0.65–1.37) | (0.58–1.15) | (0.79–2.13) | (0.67–1.51) | (0.48–1.40) |
| Count score of cognitive activities (from 0 to 11 cognitive activities) | 0.80*** | 0.80*** | 0.86** | 0.77*** | 0.74*** | 0.82** |
|  | (0.74–0.86) | (0.73–0.88) | (0.78–0.94) | (0.69–0.86) | (0.67–0.83) | (0.71–0.93) |
| Loneliness (from 1 = never/almost never to 4 = almost or almost always, whereby higher values reflect higher loneliness) | 1.42*** | 1.35* | 0.90 | 1.05 | 1.71*** | 1.48** |
|  | (1.16–1.74) | (1.07–1.70) | (0.68–1.21) | (0.71–1.56) | (1.31–2.24) | (1.10–1.98) |
| Meaning in life: - Neither (Ref.: No) | 0.89 | 0.38* | 0.45 | 0.49 | 1.14 | 0.41+ |
|  | (0.40–2.00) | (0.17–0.86) | (0.15–1.34) | (0.15–1.64) | (0.41–3.19) | (0.16–1.04) |
| - Yes | 0.92 | 0.25*** | 0.45+ | 0.31* | 1.28 | 0.23*** |
|  | (0.48–1.78) | (0.12–0.50) | (0.17–1.16) | (0.10–0.91) | (0.54–3.03) | (0.10–0.50) |
| Self-rated health (ranging from 1 = very bad to 4 = very good) | 1.09 | 1.39* | 0.91 | 0.81 | 1.29 | 1.80** |
|  | (0.87–1.37) | (1.02–1.89) | (0.67–1.25) | (0.53–1.24) | (0.92–1.80) | (1.18–2.74) |
| Count score of chronic conditions (based on 21 chronic conditions) | 0.99 | 0.99 | 0.99 | 1.13* | 1.00 | 0.90* |
|  | (0.93–1.04) | (0.92–1.06) | (0.91–1.07) | (1.03–1.24) | (0.92–1.08) | (0.82–0.99) |
| Functional impairment (ADL; ranging from 0 to 2, whereby higher values reflect higher functional impairment) | 2.37*** | 8.40*** | 2.36* | 2.07* | 2.10** | 18.18*** |
|  | (1.49–3.76) | (5.26–13.41) | (1.18–4.72) | (1.12–3.82) | (1.22–3.63) | (9.90–33.40) |
| Constant | 1.08 | 0.89 | 6.06* | 5.23+ | 0.11** | 0.17+ |
|  | (0.34–3.41) | (0.23–3.37) | (1.22–30.21) | (0.76–35.90) | (0.02–0.55) | (0.02–1.14) |
|  |  |  |  |  |  |  |
| Observations | 2,560 | | 1,322  0.12 | | 1,238 | |
| Pseudo-R² | 0.18 | |  |  | 0.28 | |

Relative risk ratios are shown; 95% CI in parentheses; *** p < 0.001, ** p < 0.01, * p < 0.05; sampling weights were applied (also adjusted for sample cells, which are utilized for the stratification of the secondary sampling unit).

Supplementary Table 2. Factors associated with probable mild cognitive impairment and probable dementia. Findings of multinomial logistic regressions (base outcome: individuals not being cognitively impaired) – without functional impairment

| Independent variables | Probable mild cognitive impairment | Probable dementia | Probable mild cognitive impairment – among men | Probable dementia – among men | Probable mild cognitive impairment – among women | Probable dementia – among women |
| --- | --- | --- | --- | --- | --- | --- |
|  |  |  |  |  |  |  |
| Sex: Women (Ref.: Men) | 0.24*** | 0.39** |  |  |  |  |
|  | (0.16–0.37) | (0.21–0.74) |  |  |  |  |
| Age group: − 85 years to 89 years (Ref.: 80 to 84 years) | 2.12*** | 2.12* | 1.06 | 1.54+ | 2.11*** | 2.27** |
|  | (1.40–3.21) | (1.18–3.82) | (0.72–1.56) | (0.95–2.50) | (1.39–3.22) | (1.25–4.15) |
| − 90 years and over | 5.89*** | 3.37** | 1.50 | 3.08*** | 6.25*** | 3.25** |
|  | (3.36–10.32) | (1.62–7.00) | (0.89–2.52) | (1.68–5.64) | (3.54–11.05) | (1.52–6.95) |
| Marital status: Married, living together with spouse/Divorced/Widowed/Single (Ref.: Married) | 1.14 | 1.01 | 1.28 | 1.50 | 1.09 | 0.75 |
|  | (0.84–1.55) | (0.65–1.57) | (0.85–1.90) | (0.87–2.58) | (0.68–1.74) | (0.41–1.37) |
| Educational level (ISCED-2011): - medium (Ref.: low) | 0.67* | 0.45*** | 0.57 | 0.26** | 0.59* | 0.49* |
|  | (0.45–0.98) | (0.28–0.71) | (0.22–1.47) | (0.11–0.61) | (0.37–0.92) | (0.28–0.84) |
| - high | 0.55** | 0.28*** | 0.34* | 0.19*** | 0.85 | 0.25** |
|  | (0.36–0.86) | (0.15–0.52) | (0.13–0.88) | (0.07–0.50) | (0.49–1.47) | (0.09–0.69) |
| Region: East Germany (Ref.: West Germany) | 1.19 | 1.16 | 1.25 | 0.61 | 1.08 | 1.72+ |
|  | (0.90–1.58) | (0.74–1.82) | (0.85–1.84) | (0.33–1.12) | (0.70–1.67) | (0.98–3.01) |
| Presence of sports activity: Yes (Ref.: No) | 0.83 | 0.64* | 0.77 | 1.23 | 0.88 | 0.42** |
|  | (0.63–1.08) | (0.42–0.97) | (0.55–1.10) | (0.76–2.00) | (0.60–1.30) | (0.24–0.77) |
| Count score of cognitive activities (from 0 to 11 cognitive activities) | 0.79*** | 0.74*** | 0.84*** | 0.75*** | 0.74*** | 0.73*** |
|  | (0.73–0.85) | (0.68–0.82) | (0.76–0.92) | (0.67–0.84) | (0.66–0.82) | (0.64–0.85) |
| Loneliness (from 1 = never/almost never to 4 = almost or almost always, whereby higher values reflect higher loneliness) | 1.45*** | 1.41** | 0.92 | 1.07 | 1.78*** | 1.59** |
|  | (1.18–1.78) | (1.11–1.79) | (0.69–1.23) | (0.73–1.57) | (1.35–2.35) | (1.17–2.16) |
| Meaning in life: - Neither (Ref.: No) | 0.87 | 0.44+ | 0.54 | 0.58 | 1.04 | 0.45 |
|  | (0.38–1.98) | (0.18–1.06) | (0.17–1.69) | (0.18–1.84) | (0.37–2.94) | (0.16–1.29) |
| - Yes | 0.83 | 0.19*** | 0.43+ | 0.30* | 1.13 | 0.16*** |
|  | (0.42–1.61) | (0.08–0.42) | (0.18–1.08) | (0.10–0.88) | (0.47–2.76) | (0.06–0.43) |
| Self-rated health (ranging from 1 = very bad to 4 = very good) | 0.97 | 0.92 | 0.78 | 0.71 | 1.19 | 1.07 |
|  | (0.78–1.22) | (0.68–1.24) | (0.58–1.05) | (0.47–1.08) | (0.85–1.66) | (0.70–1.61) |
| Count score of chronic conditions (based on 21 chronic conditions) | 1.01 | 1.02 | 1.01 | 1.15** | 1.02 | 0.96 |
|  | (0.95–1.06) | (0.95–1.10) | (0.93–1.09) | (1.05–1.26) | (0.94–1.10) | (0.86–1.06) |
| Constant | 1.84 | 7.99** | 12.02** | 9.16* | 0.17* | 3.73 |
|  | (0.58–5.79) | (2.13–29.94) | (2.63–54.86) | (1.46–57.52) | (0.04–0.80) | (0.68–20.55) |
|  |  |  |  |  |  |  |
| Observations | 2,560 | | 1,322  0.11 | | 1,238 | |
| Pseudo-R² | 0.14 | |  |  | 0.19 | |

Relative risk ratios are shown; 95% CI in parentheses; *** p < 0.001, ** p < 0.01, * p < 0.05; sampling weights were applied (also adjusted for sample cells, which are utilized for the stratification of the secondary sampling unit).

Supplementary Table 3. Factors associated with probable mild cognitive impairment and probable dementia. Findings of multinomial logistic regressions (base outcome: individuals not being cognitively impaired) – without functional impairment and lifestyle factors

| Independent variables | Probable mild cognitive impairment | Probable dementia | Probable mild cognitive impairment – among men | Probable dementia – among men | Probable mild cognitive impairment – among women | Probable dementia – among women |
| --- | --- | --- | --- | --- | --- | --- |
|  |  |  |  |  |  |  |
| Sex: Women (Ref.: Men) | 0.24*** | 0.24*** |  |  |  |  |
|  | (0.16–0.36) | (0.16–0.36) |  |  |  |  |
| Age group: − 85 years to 89 years (Ref.: 80 to 84 years) | 2.07*** | 2.07*** | 1.10 | 1.59+ | 2.05*** | 2.68*** |
|  | (1.35–3.16) | (1.35–3.16) | (0.76–1.59) | (0.99–2.57) | (1.34–3.14) | (1.51–4.79) |
| − 90 years and over | 6.03*** | 6.03*** | 1.61+ | 3.21*** | 6.25*** | 4.36*** |
|  | (3.49–10.41) | (3.49–10.41) | (0.96–2.71) | (1.76–5.88) | (3.63–10.75) | (2.23–8.49) |
| Marital status: Married, living together with spouse/Divorced/Widowed/Single (Ref.: Married) | 1.13 | 1.13 | 1.24 | 1.38 | 1.11 | 0.78 |
|  | (0.84–1.52) | (0.84–1.52) | (0.85–1.83) | (0.83–2.31) | (0.71–1.75) | (0.42–1.42) |
| Educational level (ISCED-2011): - medium (Ref.: low) | 0.56** | 0.56** | 0.52 | 0.24*** | 0.49** | 0.43** |
|  | (0.37–0.84) | (0.37–0.84) | (0.21–1.27) | (0.11–0.56) | (0.31–0.78) | (0.25–0.73) |
| - high | 0.39*** | 0.39*** | 0.26** | 0.15*** | 0.56* | 0.16*** |
|  | (0.25–0.60) | (0.25–0.60) | (0.11–0.65) | (0.06–0.37) | (0.33–0.97) | (0.06–0.45) |
| Region: East Germany (Ref.: West Germany) | 1.25 | 1.25 | 1.37+ | 0.66 | 1.10 | 1.76* |
|  | (0.96–1.63) | (0.96–1.63) | (0.94–1.99) | (0.37–1.20) | (0.73–1.64) | (1.01–3.07) |
| Loneliness (from 1 = never/almost never to 4 = almost or almost always, whereby higher values reflect higher loneliness) | 1.47*** | 1.44** | 0.95 | 1.11 | 1.79*** | 1.57** |
|  | (1.20–1.80) | (1.14–1.83) | (0.72–1.26) | (0.78–1.59) | (1.38–2.33) | (1.17–2.11) |
| Meaning in life: - Neither (Ref.: No) | 0.91 | 0.43* | 0.49 | 0.49 | 1.20 | 0.44+ |
|  | (0.43–1.96) | (0.19–0.97) | (0.15–1.53) | (0.16–1.49) | (0.46–3.09) | (0.18–1.12) |
| - Yes | 0.82 | 0.17*** | 0.38* | 0.27* | 1.17 | 0.14*** |
|  | (0.43–1.54) | (0.08–0.35) | (0.15–0.95) | (0.10–0.75) | (0.50–2.70) | (0.06–0.32) |
| Self-rated health (ranging from 1 = very bad to 4 = very good) | 0.88 | 0.80 | 0.71* | 0.64* | 1.04 | 0.92 |
|  | (0.71–1.08) | (0.60–1.06) | (0.53–0.93) | (0.44–0.94) | (0.76–1.43) | (0.63–1.33) |
| Count score of chronic conditions (based on 21 chronic conditions) | 1.00 | 1.02 | 1.01 | 1.15** | 1.00 | 0.95 |
|  | (0.95–1.05) | (0.95–1.10) | (0.94–1.09) | (1.05–1.26) | (0.93–1.08) | (0.87–1.05) |
| Constant | 1.00 | 3.38+ | 7.69** | 5.32+ | 0.08** | 1.17 |
|  | (0.32–3.10) | (0.91–12.56) | (1.74–34.07) | (0.90–31.39) | (0.02–0.37) | (0.24–5.80) |
|  |  |  |  |  |  |  |
| Observations | 2,596 | | 1,336  0.09 | | 1,260 | |
| Pseudo-R² | 0.11 | |  |  | 0.15 | |

Relative risk ratios are shown; 95% CI in parentheses; *** p < 0.001, ** p < 0.01, * p < 0.05; sampling weights were applied (also adjusted for sample cells, which are utilized for the stratification of the secondary sampling unit).
